# Project automation in CCP4 Cloud: Enabling customization and high‐throughput efficiency

**DOI:** 10.1002/pro.70176

**Published:** 2025-05-24

**Authors:** Eugene Krissinel, Maria Fando, Oleg Kovalevskiy, Ronan Keegan, Jools Wills, Andrey Lebedev, Ville Uski, Charles Ballard

**Affiliations:** ^1^ Research Complex at Harwell, Scientific Computing Department Science and Technology Facilities Council Harwell UK

**Keywords:** automatic workflows, computational cloud, macromolecular crystallography, structure solution

## Abstract

The rapid advancement of automatic structure solution methods, driven by the availability of high‐quality predicted structures from AlphaFold and the growing adoption of multi‐crystal and serial experiments, has created a pressing need for streamlining routine operations, automating structure solution projects, and efficiently handling large volumes of data. Modern software solutions must be both robust and user‐friendly, supporting manual workflows while enabling high‐throughput operations to keep pace with the high data collection rates of modern beamlines. Here, we present new developments in CCP4 Cloud that address these challenges by providing predefined and customizable automatic workflows, which can be seamlessly integrated with experimental facilities, offering a powerful solution for modern macromolecular crystallography. CCP4 Cloud is available as a public service at https://cloud.ccp4.ac.uk.

## 1 INTRODUCTION

Solving macromolecular structures is often a complex task that resists straightforward algorithmic solutions. Common challenges include poor diffraction, crystal defects, low resolution, weak anomalous signals, and—particularly in molecular replacement—the lack of close structural templates. However, the latter issue has significantly diminished with the advent of AlphaFold 2 (Jumper et al. 2021), which made it possible to generate high‐quality structural models for almost all UniProt (The UniProt Consortium 2025) sequences, now available in the AlphaFold Database (AFDB) (Varadi et al. 2024). Recent studies indicate that nearly 97% of structures deposited in the Protein Data Bank (PDB) (Berman et al. 2003; Burley et al. 2025) since AlphaFold's introduction can be solved through molecular replacement using AFDB models or AlphaFold‐derived predictions (Keegan et al. 2024; Terwilliger et al. 2023).

The success of AlphaFold‐assisted molecular replacement (MR) suggests that a fully automated approach to structure solution is feasible in most cases. Yet, manual intervention is often required for specific challenges, such as managing unique structural conformations or assembling multi‐domain models for macromolecular complexes. Once a structural model is built and refined, along with the established structure solution protocol, it serves as a foundation for efficient structure determination in subsequent experimental studies of target protein, as well as for applications such as high‐throughput screening and serial crystallography.

This suggests that in macromolecular crystallography (MX), computational projects can be categorized into two main groups:

- **New Structure Solutions**: These projects involve solving novel structures, requiring multiple iterations, non‐trivial data manipulations, and innovative strategies. They present both scientific and technical challenges.
- **Established Protocols**: These projects follow pre‐established structure solution pipelines, often based on MR, with customizations such as specific refinement procedures, complex model handling, and ligand fitting.

Traditionally, graphical user interfaces (GUIs) like CCP4i (Berman et al. 2003; Potterton et al. 2003; Potterton et al. 2018), Phenix (Liebschner et al. 2019), and HKL‐3000 (Minor et al. 2006) have supported the first group by facilitating flexible manipulations, accelerating the discovery of suitable protocols. For the second group, particularly in high‐throughput environments, low‐level scripting is more effective for creating efficient pipelines like Xia‐2 (Winter 2010), MrBUMP (Keegan et al. 2011; Keegan et al. 2018; Keegan and Winn 2008), MoRDa (Vagin and Lebedev 2015), Dimple (Wojdyr et al. 2013), Crank‐2 (Skubák et al. 2018) and similar. GUIs are less suited to these repetitive workflows due to their manual nature, while scripting can be cumbersome for managing the complexity and data bookkeeping inherent to new structure solution projects.

We present recent advancements in CCP4 Cloud (Krissinel et al. 2022) (CCP4: Collaborative Computational Project Number 4 in Protein Crystallography), an online system for MX calculations built on the CCP4 Software Suite (Agirre et al. 2023), that bridges the gap between the two main groups of MX projects by introducing high‐level automation on top of the established web GUI. This feature streamlines repetitive tasks in structure solution projects and enables fully automated structure determination using established protocols.

Additionally, the system includes tools for seamlessly transferring data and structure solution protocols from the data collection point to the Cloud, enabling complete automation in repetitive MX experiments. The proposed approach combines ease of implementation and customization, minimal maintenance requirements, and a robust project management and bookkeeping framework within CCP4 Cloud.

CCP4 Cloud is accessible as a public service at https://cloud.ccp4.ac.uk and is also available for in‐house installation as part of the CCP4 Software Suite.

## 2 RESULTS

### 2.1 Predefined workflows in CCP4 Cloud

CCP4 Cloud workflows are built on a foundation of fundamental tasks to automate routinely repeated actions in structure determination. For uncomplicated datasets—such as those with resolution better than 2–2.5 Å, no significant crystal pathologies, and confident space group determination—these automated workflows can often produce solutions that are nearly final. Accordingly, all predefined workflows conclude with the generation of a PDB Validation Report (Read et al. 2011), enabling assessment of the final model's quality. Each task within a workflow is represented in the project tree exactly as if it had been initiated manually by a user. Moreover, every task can be cloned and re‐run with modified parameters, automatically creating an alternative branch in the project tree. This flexibility allows users to easily explore different approaches to structure determination, potentially achieving results with improved metrics. Below, we provide a brief overview of the predefined CCP4 Cloud workflows that are available out of the box.

#### 2.1.1 auto‐MR: Molecular replacement with MrBUMP or MoRDa

**FIGURE 1:**
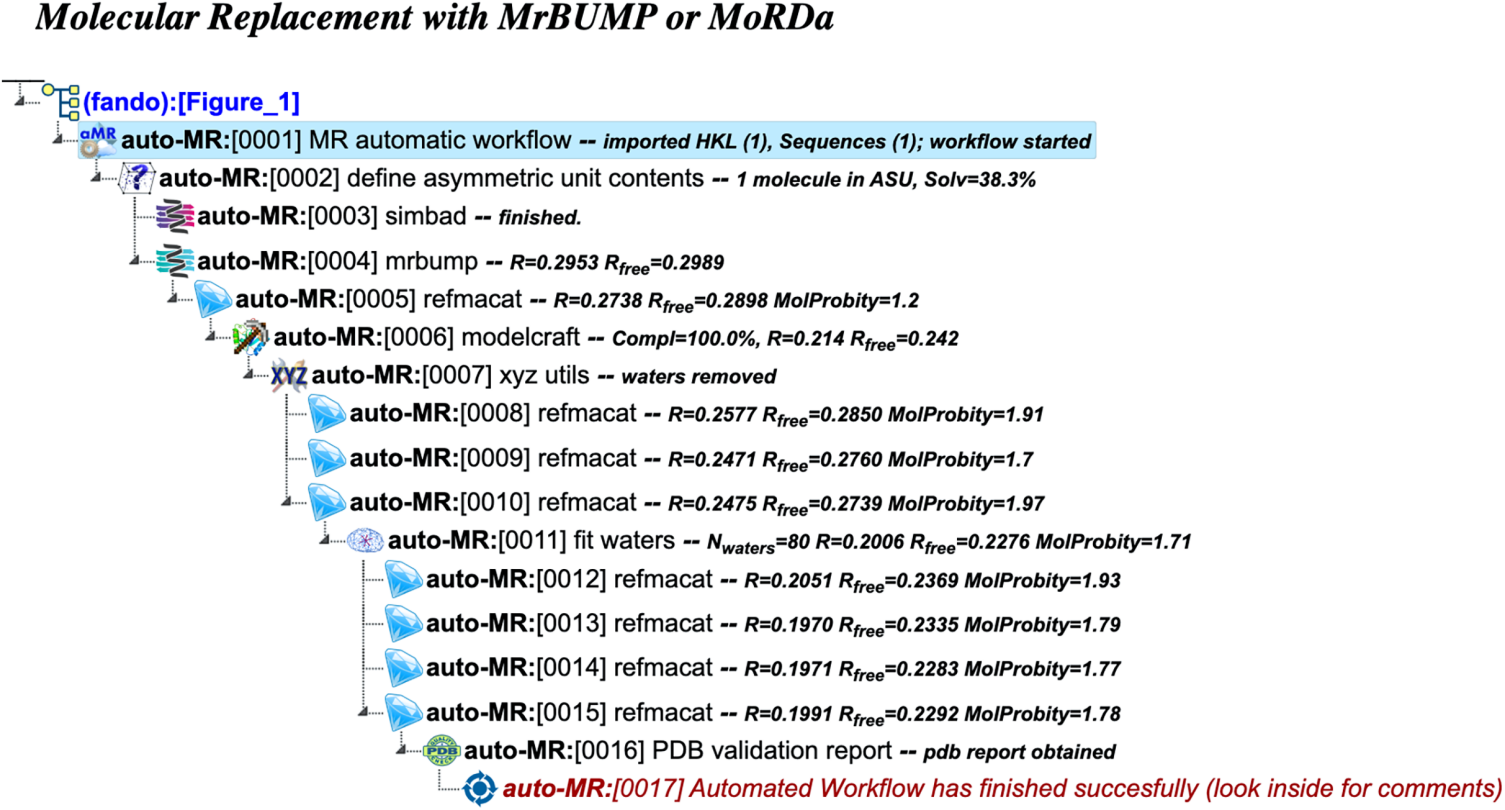
Project tree generated by the automated workflow “Molecular replacement using MrBump or MoRDa” (auto‐MR). The figure illustrates a specific example based on particular input data and does not represent the complete workflow flowchart. The full project can be examined in the CCP4 Cloud Archive under the identifier PS25F1: https://cloud.ccp4.ac.uk/archive/access.html?id=CCP4‐PS25F1.

This workflow streamlines the use of structural databases for Molecular Replacement (Figure 1). It requires input of merged or unmerged reflection data, macromolecular sequence, and, optionally, a ligand description. If unmerged data is provided, the workflow employs Aimless (Evans and Murshudov 2013) to scale and merge the data and then estimates the asymmetric unit content. Next, it uses the Simbad software (Simpkin et al. 2018) to check whether a quick solution can be obtained from the analysis of the rotation function and to determine whether the data correspond to a contaminant. Phase determination begins with MrBUMP, which automatically searches for structural templates in the PDB. If unsuccessful, the workflow switches to MoRDa, which uses its curated database of structural domains to attempt an alternative solution. Once the phase problem is resolved, the structure is rebuilt using Modelcraft (Bond and Cowtan 2022) in order to remove differences between the used structural homolog and the target sequence. If a ligand description is given, the workflow generates ligand structures and attempts to fit them in a suitable density feature using Coot (Emsley et al. 2010) software. Finally, water molecules are added to appropriate density blobs using Coot's FindWaters (Emsley et al. 2012) utility, and the structure undergoes iterative refinement using methods from the auto‐REL workflow (see below).

#### 2.1.2 af‐MR: Molecular replacement with an AlphaFold model

**FIGURE 2:**
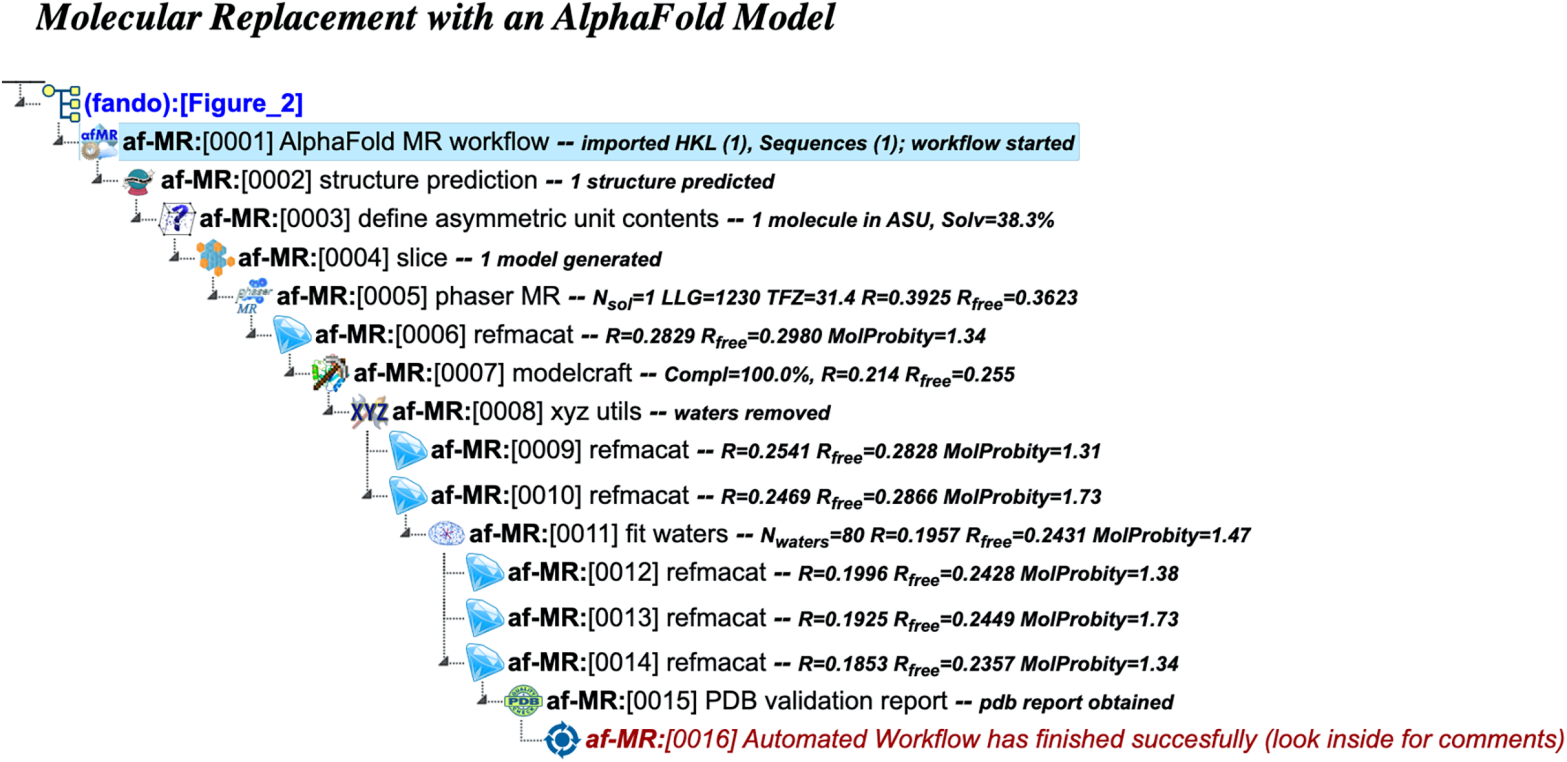
Project tree generated by the automated workflow “Molecular replacement with a generated AlphaFold model” (af‐MR). The figure illustrates a specific example based on particular input data and does not represent the complete workflow flowchart. The full project can be examined in the CCP4 Cloud Archive under the identifier PS25F2: https://cloud.ccp4.ac.uk/archive/access.html?id=CCP4‐PS25F2.

This workflow simplifies the integration of AlphaFold predictions into molecular replacement, providing an efficient alternative to traditional methods (Figure 2). It requires the same input as the auto‐MR workflow: merged or unmerged reflection data, a macromolecular sequence, and an optional ligand description. While it follows the same principal stages of structure solution, this workflow replaces the use of MrBUMP or MoRDa with a tailored process involving AlphaFold. The structural template is generated directly using AlphaFold 2 prediction software via Colabfold (Mirdita et al. 2022), with low‐confidence regions pruned and residue pLDDT confidence values converted to B‐factor estimates using Slice (Simpkin et al. 2025). The resulting refined model is then employed for molecular replacement with Phaser (McCoy et al. 2007), streamlining the process of phase determination.

#### 2.1.3 simple‐MR: Simple molecular replacement with Search model

**FIGURE 3:**
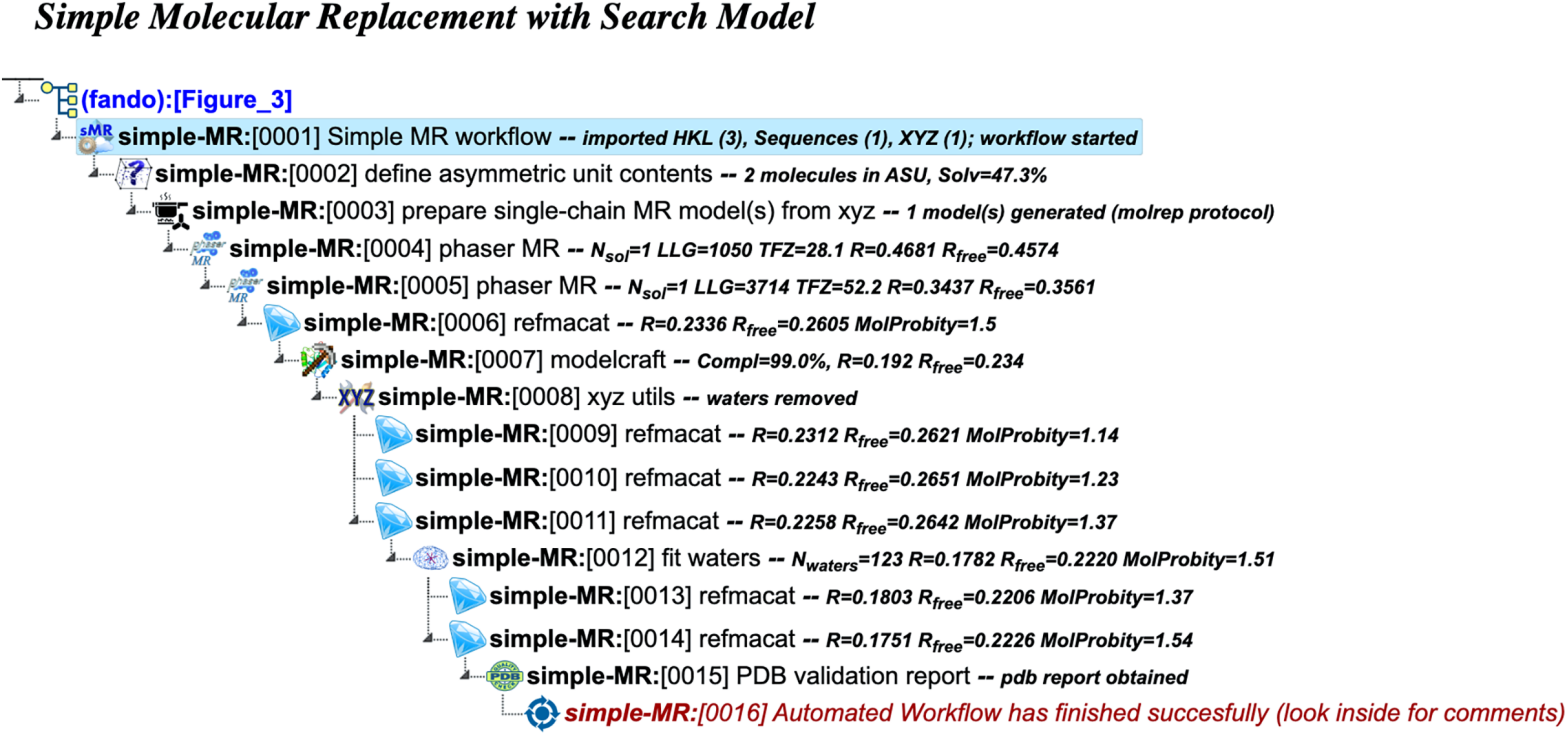
Project tree generated by the automated workflow “Molecular replacement using a known structure model” (simple‐MR). The figure illustrates a specific example based on particular input data and does not represent the complete workflow flowchart. The full project can be examined in the CCP4 Cloud Archive under the identifier PS25F3: https://cloud.ccp4.ac.uk/archive/access.html?id=CCP4‐PS25F3.

This workflow is designed for situations where a reliable structural template is available, providing a straightforward approach to molecular replacement (Figure 3). It requires the same input as the above workflows, along with a PDB or mmCIF‐formatted file (Westbrook et al. 2022) containing the coordinates of the structural template. The process follows the same overall pathway as the af‐MR workflow but uses the supplied template instead of generating a model with AlphaFold. The template is employed for phase determination using Phaser. Since the workflow assumes that the template's backbone, though not necessarily its sequence, closely resembles the target structure, Molrep (Vagin and Teplyakov 1997) is used to prune mismatched side‐chains before the phasing step.

#### 2.1.4 auto‐DPLMR: Dimple molecular replacement

**FIGURE 4:**
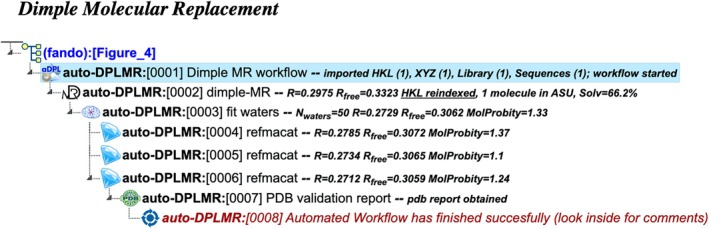
Project tree generated by the automated workflow “Fast molecular replacement via Dimple with a high‐homology model” (auto‐DPLMR). The figure illustrates a specific example based on particular input data and does not represent the complete workflow flowchart. The full project can be examined in the CCP4 Cloud Archive under the identifier PS25F4: https://cloud.ccp4.ac.uk/archive/access.html?id=CCP4‐PS25F4.

This workflow is a specialized version of the simple‐MR workflow, tailored for cases where the supplied structural homolog is an exact match to the target protein (Figure 4). Such scenarios are common in fragment screening experiments (de Souza Neto et al. 2024; Lima et al. 2020), where the template typically exhibits 100% sequence identity with the target. The perfect homology allows the workflow to significantly reduce the computational overhead by utilizing the highly efficient Dimple pipeline (DIMPLE‐MX Pipeline 2025) for molecular replacement. Unlike other workflows, this approach omits additional model building and refinement steps, as Dimple itself incorporates the necessary level of refinement.

#### 2.1.5 auto‐EP: Automated experimental phasing with Crank‐2

**FIGURE 5:**
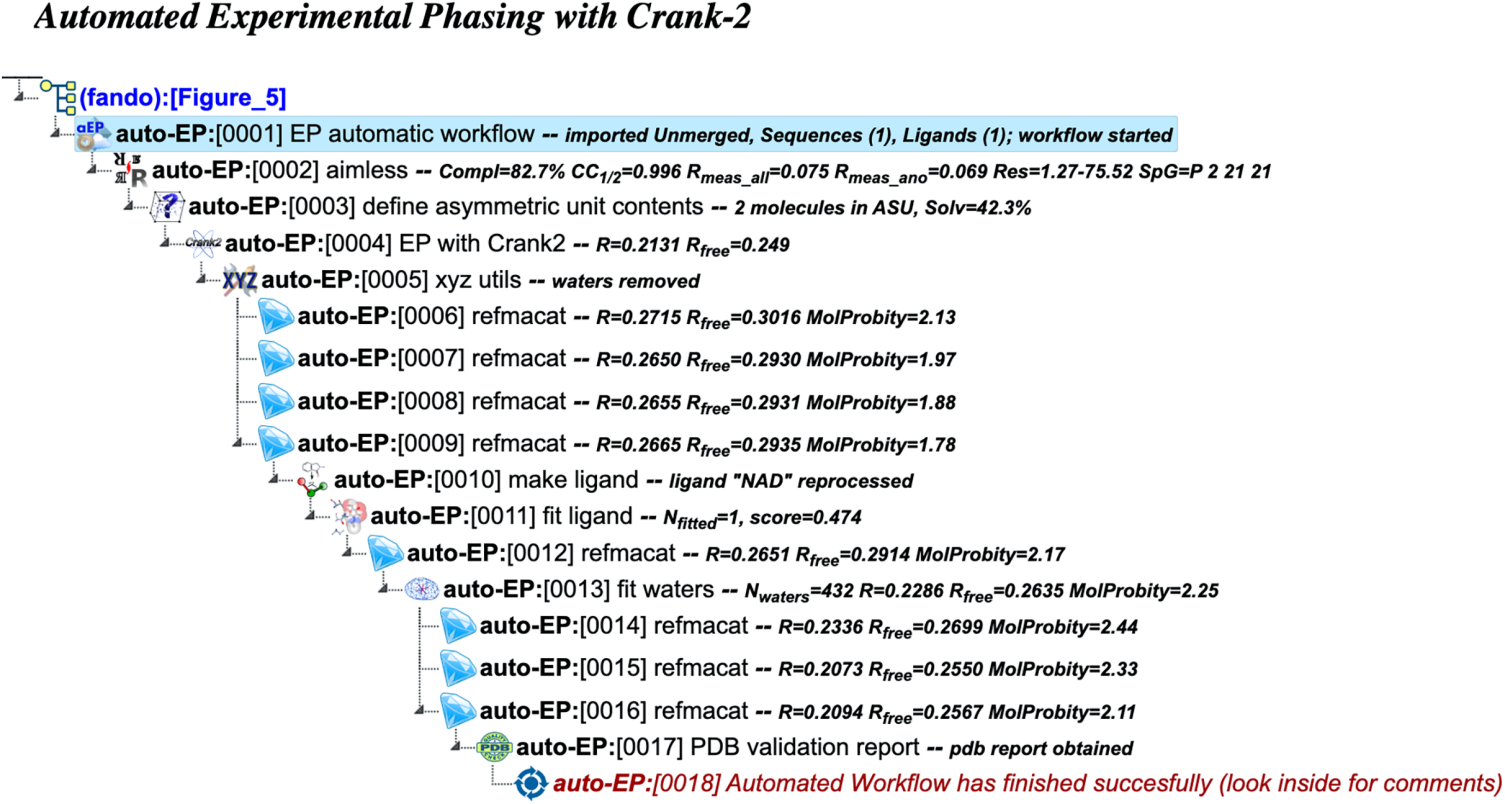
Project tree generated by the automated workflow “Experimental phasing with Crank‐2” (auto‐EP). The figure illustrates a specific example based on particular input data and does not represent the complete workflow flowchart. The full project can be examined in the CCP4 Cloud Archive under the identifier PS25F5: https://cloud.ccp4.ac.uk/archive/access.html?id=CCP4‐PS25F5.

This workflow simplifies the process of experimental phasing by utilizing the Crank‐2 software (Skubák et al. 2018) (Figure 5), an automated pipeline for solving macromolecular structures by experimental phasing. The workflow requires input data consisting of merged or unmerged reflections with an anomalous signal, a macromolecular sequence, the atom type of the primary anomalous scatterers, and an optional ligand description. If unmerged data is provided, the workflow first merges and scales the reflections with Aimless, and then estimates the content of the asymmetric unit. It then starts Crank‐2, which identifies the anomalous substructure and uses it to solve the phase problem. As Crank‐2 includes model building and refinement, these steps are omitted from the workflow. Following Crank‐2, the workflow proceeds with ligand and solvent fitting, followed by iterative refinement similar to the auto‐REL workflow (see below).

#### 2.1.6 auto‐DPL: Dimple refinement and ligand fitting

**FIGURE 6:**
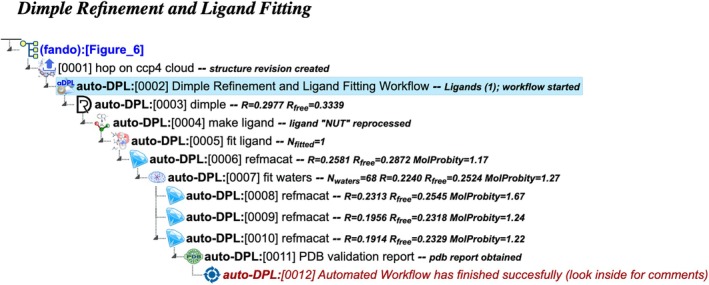
Project tree generated by the automated workflow “Refinement and ligand fitting using Dimple” (auto‐DPL), initiated from Job 0002 using a partially solved structure imported in Job 0001. Alternatively, it could have been started, for example, after MR project as illustrated in Figure 7. The figure illustrates a specific example based on particular input data and does not represent the complete workflow flowchart. The full project can be examined in the CCP4 Cloud Archive under the identifier PS25F6: https://cloud.ccp4.ac.uk/archive/access.html?id=CCP4‐PS25F6.

This workflow utilizes Dimple software to perform structure refinement, including rigid‐body adjustments (Figure 6). It requires input in the form of a phased structure and, optionally, a ligand description. In CCP4 Cloud, a phased structure encompasses merged reflection data and a correctly positioned structural model, supplied as a unified data object within the framework of a structure revision—a universal internal data type used across CCP4 Cloud projects. The workflow begins with optional re‐positioning and refinement of the structural model using Dimple. If a ligand description is provided, the workflow attempts to fit the ligand into the electron density, followed by the addition of solvent molecules. Iterative refinement is then performed to further optimize the structure (see details below).

#### 2.1.7 auto‐REL: Automated refinement and ligand fitting

**FIGURE 7:**
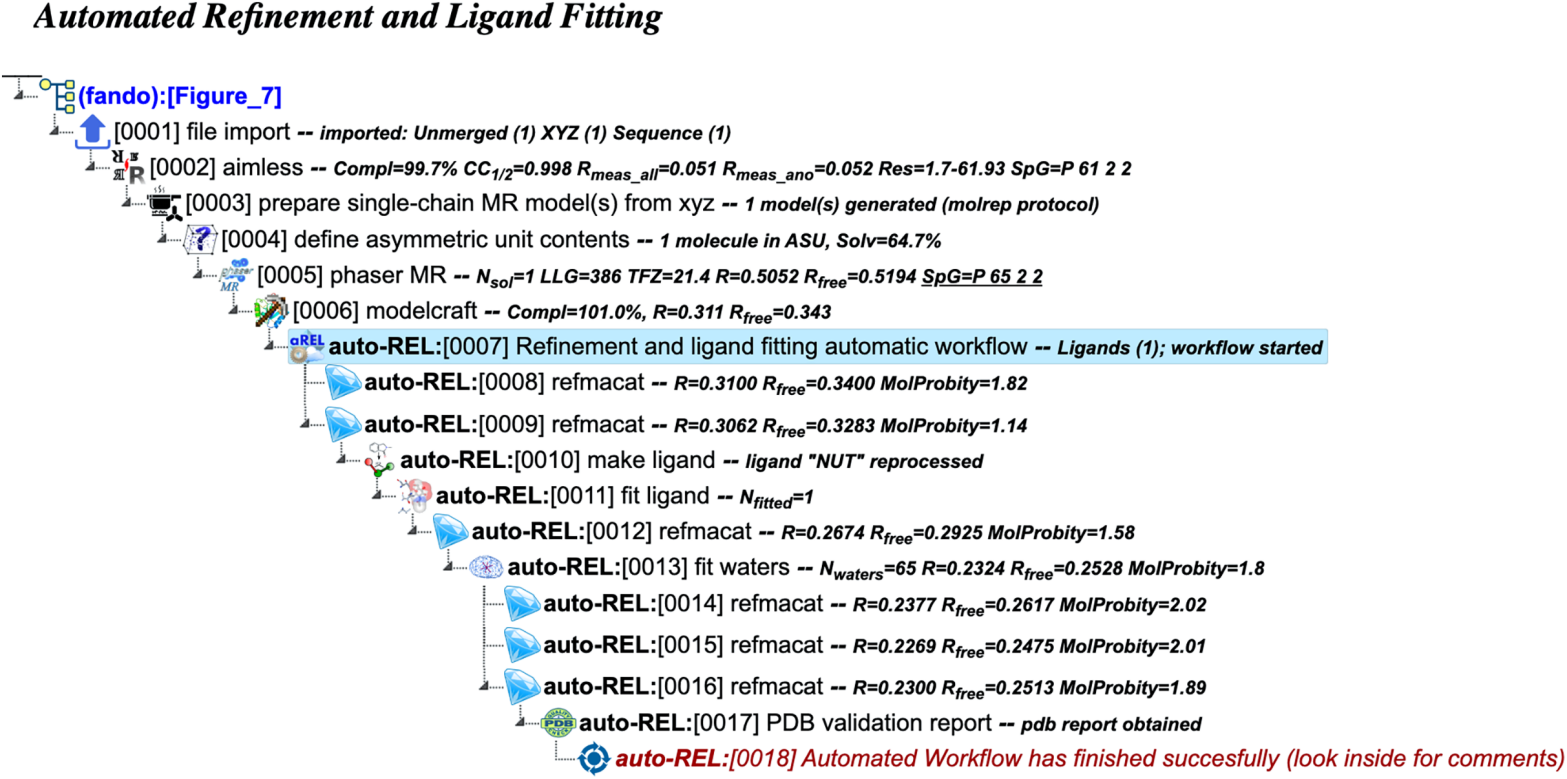
Project tree generated by the automated workflow “Refinement and ligand fitting using Refmacat” (auto‐REL), initiated from Job 0007 using results from a manually developed MR project upstream (Jobs 0001‐0006). The figure illustrates a specific example based on particular input data and does not represent the complete workflow flowchart. The full project can be examined in the CCP4 Cloud Archive under the identifier PS25F7: https://cloud.ccp4.ac.uk/archive/access.html?id=CCP4‐PS25F7.

This workflow automates the refinement of structures and the fitting of ligand and solvent molecules—key tasks in structure completion (Figure 7). A distinguishing feature of this workflow, based on REFMAC5/Refmacat (Murshudov et al. 2011; Yamashita et al. 2023) and Coot software, is its ability to optimize refinement parameters iteratively. In CCP4 Cloud, tasks are enhanced with verdict sections, which provide a detailed quality analysis of the resulting structure and suggestions for further optimization. The workflow integrates these recommendations automatically, eliminating the need for manual intervention during the refinement process: using REFMAC5 verdict, the workflow identifies opportunities for improvement and automatically applies the suggested parameters in subsequent (cloned) iterations. This process typically converges after 4–6 iterations, ensuring optimal refinement. Similar to the auto‐DPL workflow, this approach requires a phased structure and an optional ligand description as input.

### 2.2 Custom workflows

While the predefined workflows in CCP4 Cloud address most common scenarios in structure determination, practical cases may benefit from customization. For instance, tailoring individual task parameters can significantly reduce CPU time, especially when optimized for a specific target structure in serial experiments. Similarly, customization may involve adjusting workflows, for example, to bring multiple datasets to a common resolution range for comparative analysis. In some cases, steps that are generally justified in broader contexts might be unnecessary for a particular experiment and can be omitted. Alternatively, compatible tasks offering better performance under specific conditions may replace default steps. CCP4 Cloud accommodates these needs by allowing users to define and execute custom workflows, just like any other task. This flexibility ensures that workflows can be adapted to meet the unique requirements of diverse research scenarios.

Custom workflows within CCP4 Cloud are created using WScript (Workflow Script), a specialized scripting language tailored to the unique requirements of project development in the system. The introduction of WScript addresses critical security concerns, particularly in public‐facing services, where the use of traditional scripting languages such as Python can introduce vulnerabilities. WScript scripts are structured into three main sections.

#### 2.2.1 Workflow header

This section includes metadata and configuration details, such as the workflow's short name, title, keywords, brief description, and a suggested icon for the task list. These elements provide essential information to identify and organize the workflow within the system.

#### 2.2.2 Input data

This section specifies the parameters and datasets required for workflow execution, distinguishing between mandatory and optional inputs. The content of this section is rendered as a standard input panel for the corresponding CCP4 Cloud task, comprising data and parameter widgets. When the workflow is run from the top of the project tree, data widgets take the form of file upload fields; otherwise, they appear as drop‐down menus for selecting compatible data from upstream tasks. Parameter values are assigned to internal workflow variables, using the names defined in the widget descriptions.

#### 2.2.3 Workflow body

This section outlines the operational logic and procedural steps of the workflow. Each task is described in a separate subsection, which may include task‐specific parameters, references to optional data items, and conditions under which the task should be executed. WScript supports the definition of internal variables, as well as a full range of arithmetic and logical operations. It also allows for the inclusion of conditional statements to create project branches and loops to facilitate iterative refinement or exploration of parameter spaces.

**FIGURE 8:**
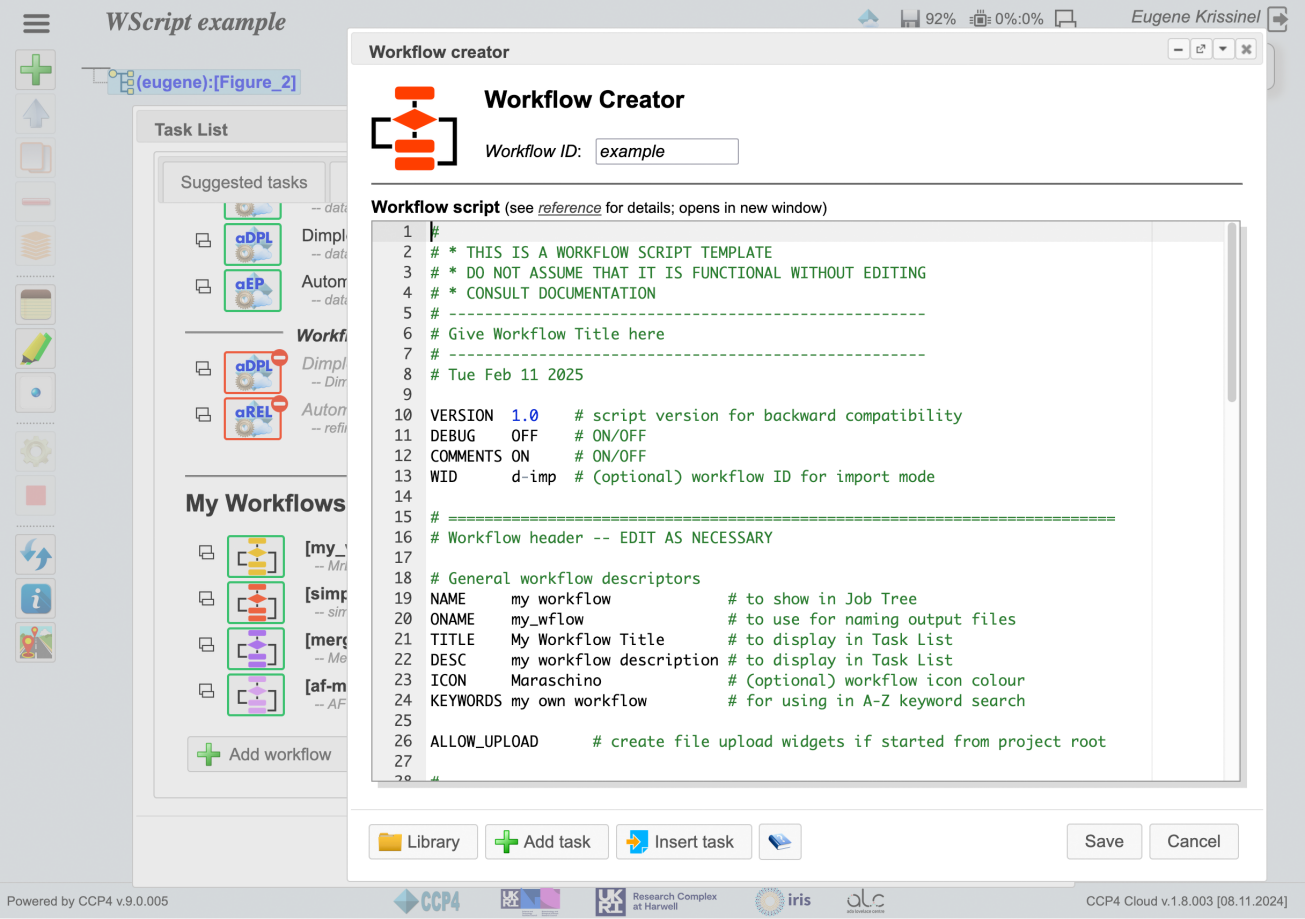
A snapshot of Workflow Creator, a graphical tool designed to assist in creating custom workflows. The workflow is defined using WScript, a specialized scripting language. Workflow Creator simplifies the addition of tasks, parameter configuration, and the integration of custom workflows into the task list for users.

Scripting with WScript is facilitated by the **Workflow Creator** (Figure 8), an integral component of CCP4 Cloud, found in the task list. The Workflow Creator offers a user‐friendly graphical interface designed to streamline the creation of WScript scripts. It assists by providing templates for all three sections of the script, ensuring consistency and accuracy. Additionally, it auto‐generates unique names for the tasks executed by the workflow and automatically maps parameter widgets to the corresponding task keywords. This functionality simplifies the scripting process, reduces errors, and enhances the overall efficiency of workflow design. A detailed description of WScript syntax and design process is found in CCP4 Cloud documentation: https://cloud.ccp4.ac.uk/manuals/html-userguide/jscofe_custom_workflows.html.

WScript scripts are stored as plain‐text files with the .wscript extension, which can be used in several ways within CCP4 Cloud. They can be utilized to create new tasks in the CCP4 Cloud task list (this is done automatically if workflow is designed with the Workflow Creator). Once added, these tasks behave like any other in the system but remain visible and accessible only to the user who created them. WScript files can also be imported along with associated data files, such as reflection data files (.mtz), sequence, or structure model files, using the general import task. If a .wscript file is detected during the import process, CCP4 Cloud automatically executes the corresponding workflow, using the imported data as input. Additionally, WScript files are compatible with the CloudRun utility (see below), allowing data uploads and the triggering of scripted workflows directly from the command line. This forms the basis of the technical framework for using the CCP4 Cloud in high‐throughput scenarios, with the flexibility to customize workflows to meet specific beamline or experimental requirements, as described in the next section.

### 2.3 Using CCP4 Cloud in high‐throughput scenarios

High‐throughput experimentation, a cornerstone of drug discovery research, depends on highly automated systems to conduct and analyze multi‐crystal experiments with minimal human intervention (Gioiello et al. 2020; Hansel et al. 2022; Schneider 2018). These systems produce data at a rapid pace, necessitating software setups capable of handling and processing datasets in a highly parallelized and automated manner. This is where the computational cloud paradigm excels.

The automated workflows described above are designed to deliver near‐final results in structure determination with minimal user input. While the processing time may not always match the speed of data production, the cloud's ability to run an effectively unlimited number of workflows simultaneously ensures that there are no additional delays in the experimental cycle. This scalability makes the cloud paradigm particularly well‐suited to the demands of high‐throughput environments.

To fully harness the power of automated workflows, CCP4 Cloud is equipped with the CloudRun utility. This command‐line tool, included in the standard CCP4 distribution, enables users to transfer data, create projects, and initiate automated workflows in a single, streamlined invocation. CloudRun operates through a set of keyword‐value statements, which can be supplied via a separate file or through the standard input stream. Key parameters for CloudRun include:

- **CCP4 Cloud server URL**: The address of the CCP4 Cloud server.
- **User login name and CloudRun** **ID**: The login credentials for the CCP4 Cloud account. CloudRun ID is a 16‐character access token, generated by a user request in CCP4 Cloud. This token provides secure access without exposing the user's login password in plain‐text files or scripts.
- **Project name, title, and folder**: A short name, optional descriptive title, and project list folder for the project where the uploaded data will be processed. If the project does not exist, it will be created automatically.
- **Workflow name**: The name of the automated workflow to be triggered upon data upload.
- **Data files and ligand descriptions**: Files containing reflection data, macromolecular sequences, template structures, ligand structures, and WScript descriptions, as needed. Expected ligands can be specified using SMILES strings (Weininger 1988) or ligand codes.

The CloudRun utility simplifies the integration of CCP4 Cloud into high‐throughput pipelines by enabling seamless automation and workflow initiation. Detailed instructions on operating CloudRun are available in the CCP4 Cloud documentation: https://cloud.ccp4.ac.uk/manuals/html-userguide/jscofe_cloudrun.html.

The CloudRun utility allows experiment‐side software to transfer produced data to CCP4 Cloud for structure determination at the end of each experimental cycle. CloudRun specifically handles processed diffraction data in the form of unmerged or merged MTZ files. This approach leverages the fact that synchrotron beamlines often run their own data processing pipelines, eliminating the need to duplicate diffraction image processing within the Cloud.

The transfer of raw diffraction images can be time‐consuming, even with high‐speed Internet, and results in significant data volumes in structure determination projects. To address this issue, CCP4 Cloud provides a dedicated storage area for diffraction images managed by a separate DataLink server. DataLink is integrated with the Globus framework for efficient file sharing and also supports direct transfer of large amounts of data from local devices to cloud storage. The details of this functionality are described elsewhere (Wills et al. 2024).

## 3 DISCUSSION AND CONCLUSIONS

The CCP4 suite is a long‐established crystallographic software project with a 46‐year history, accumulating a wide range of solutions for many crystallographic tasks. The sheer number of alternative approaches available within CCP4 highlights that there is no single ideal pathway from diffraction data to a final structure, which is also reflected in the variety of workflows presented in section 2.

The choice of a particular MR workflow depends on the availability and quality of the structural template, as well as personal preference; in challenging cases, it may be necessary to attempt multiple workflows. The most general‐purpose workflow is auto‐MR, which requires only reflection data and protein sequence. It explores multiple strategies and leverages extensive databases to maximize the chances of success. This workflow is especially well suited for solving multimeric complexes. However, it is also the slowest option. When a good structural template is already known, such as in a series of experiments involving only minor protein modifications, the simple‐MR workflow can save considerable time.

When a suitable model is not available, the af‐MR workflow offers a fast way to predict a structure and test whether it can yield a solution. Finally, auto‐DPLMR is the fastest available workflow, developed specifically for rapid phasing of isomorphous structures in high‐throughput experiments, such as fragment screening. It requires the availability of a highly similar structural template.

When none of the MR approaches yield a solution, the auto‐EP workflow can be attempted, provided the data contain a measurable anomalous signal. Although this circumstance is becoming increasingly rare, experimental phasing remains a practical method.

If the phase problem was not solved using one of the automatic workflows described above, the resulting structure can be automatically refined using one of two available workflows. The auto‐REL workflow optimizes refinement parameters through multiple refinement attempts, adds solvent molecules, and attempts to fit ligands. It is intended to save effort on routine actions typically required for improving a solution at the intermediate to final stages of the structure solution process. The auto‐DPL workflow offers functionality similar to auto‐REL, with added features such as optional rigid‐body refinement and initial space group validation. While it closely resembles the auto‐DPLMR workflow, it does not assume a complete input model and is intended for use only after the structure has been phased. This workflow is the recommended choice when importing a phased but unrefined structure into a CCP4 Cloud project.

When an automatic workflow fails or yields poor results, few corrective actions are available beyond trying alternative workflows. The first step should be to identify the source of the underperformance. In many cases, issues arise during data processing or reduction; if so, the reflection data should be reprocessed before making another attempt. Another common issue is the use of suboptimal parameters in one of the workflow tasks. In such cases, the failed job can be cloned and re‐run with adjusted parameters in “Auto” mode, which will initiate a new branch in the project that continues automatically according to the workflow algorithm. Researchers may also switch to a fully manual mode at any point in the workflow tree by adding a new task (or cloning and running an existing one in non‐“Auto” mode) and continue developing the structural solution manually using a custom pathway. To conserve computational resources, any workflow can be interrupted at any time by stopping the currently running job.

From the user's perspective, CCP4 Cloud presents itself as a web‐based GUI that facilitates the operation of various CCP4 programs and the graphical development of structure solution projects. It offers an advanced, hierarchical system for organizing multiple projects and their associated tasks, complemented by an ergonomic interface and detailed graphical task reports. Notably, CCP4 Cloud Local can also be run as a conventional GUI on any laptop or desktop computer. CCP4 Cloud projects are fully compatible across Windows, macOS, and Linux systems, and can be transferred between CCP4 Cloud servers and local installations using the project export/import functionality. Individual tasks from local projects can also be run on remote CCP4 Cloud servers, provided CCP4 version 9.0.008 or later is used.

However, the true advantage of CCP4 Cloud lies in its centralized, server‐based setups powered by high‐performance computing (HPC) facilities. These setups provide significant computational power, enabling the extensive use of automated structure solvers. They also offer seamless access to sequence and structure databases, third‐party services, and maintainable storage for user projects and data. With its server‐based design, CCP4 Cloud ensures secure, geographically independent access to user data and projects, making it far more versatile and scalable than traditional desktop GUIs.

In this work, we presented further enhancements to CCP4 Cloud, extending its capabilities beyond the typical scope of graphical user interfaces. These advancements introduce tools for fully automating the development of structure solution projects, with the ability to receive data directly from X‐ray facilities and in‐house diffractometers. Importantly, the resulting projects maintain the same structure as those developed manually, allowing users to intervene at any stage to implement improvements or explore alternative approaches as needed.

**FIGURE 9:**
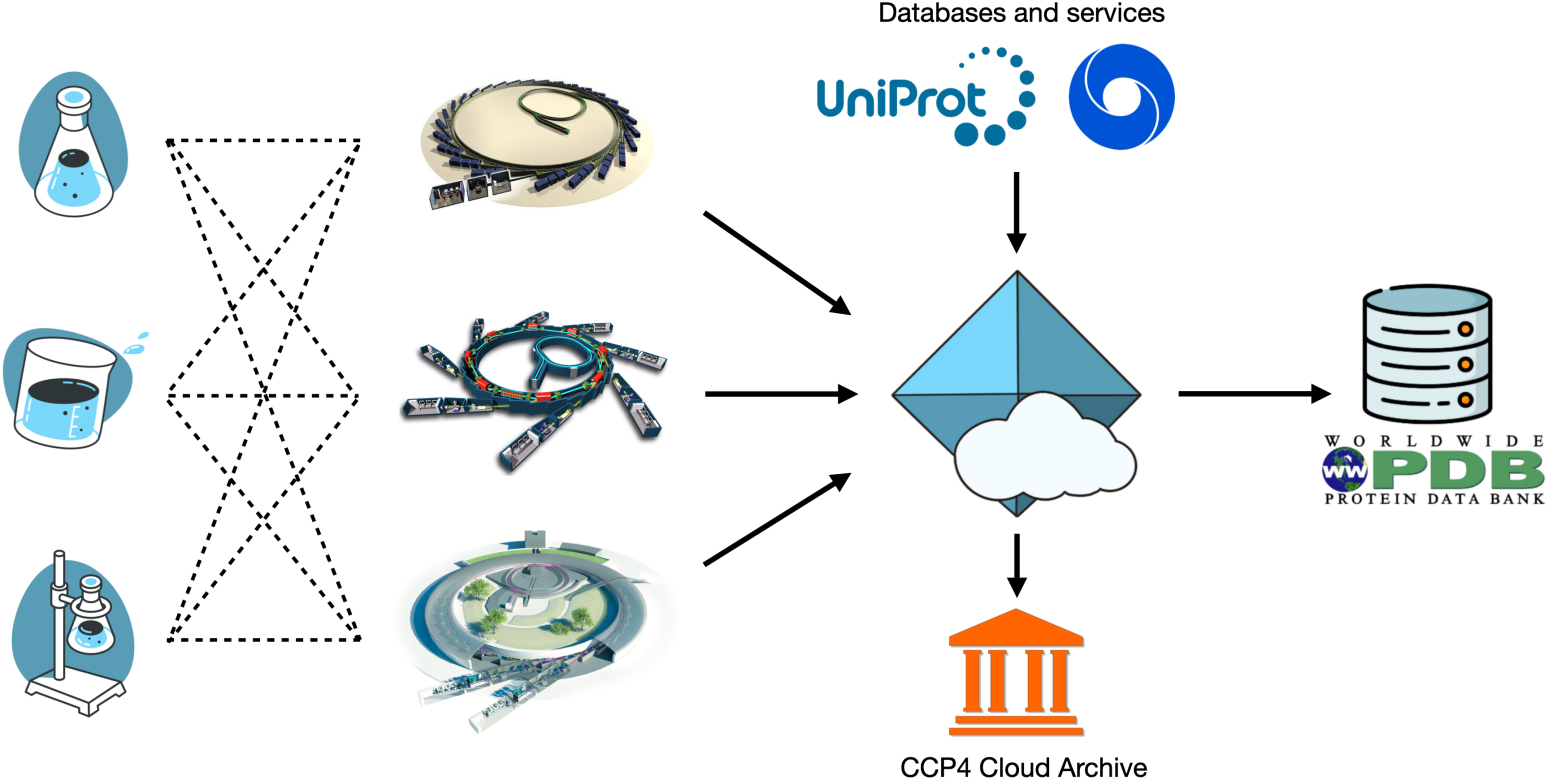
A potential operational flow for MX studies, enabled by the developments presented in this paper. Samples produced in research labs can be shipped to any XRD facility, while collected data is transferred into a centralized computational framework accessible through a single gateway. This approach assumes fully remote and online operations from sample production onward. It streamlines data processing and structure solution, facilitates the use of web‐based bioinformatics resources and services, enables efficient PDB deposition, enhances long‐term data and project retention, and optimizes computational resource utilization.

This development introduces new possibilities for streamlining MX studies. A growing trend in the field involves conducting crystallographic experiments remotely, with researchers sending samples to X‐ray facilities and then operating a beamline over the internet; alternatively, the entire data collection can be performed by the beamline team. With the enhanced capabilities of CCP4 Cloud, collected data can now be automatically transferred to the user's CCP4 Cloud account, triggering the initiation of a structure solution project. In an ideal workflow, users simply send their samples to a synchrotron and later check the results in their CCP4 Cloud accounts. From there, they can make final adjustments, obtain the PDB validation report, and proceed with PDB deposition—provided the results meet the validation criteria. Note that mmCIF deposition files become available only after completing the PDB validation task in CCP4 Cloud. Additionally, completed structure solution projects can be archived within CCP4 Cloud. This optimizes the description of computational procedures and provides a comprehensive level of detail for referees. Figure 9 illustrates the potential operational flow of MX projects enabled by this development, from data collection to publication and archiving.

In summary, we have demonstrated that the enhanced automation and direct data integration with imaging facilities in CCP4 Cloud offer significant potential for optimizing computational support in MX studies. These advancements maintain the same level of flexibility and versatility as manual operation of crystallographic software, while streamlining and automating complex workflows.

Implementing operational flows similar to the one illustrated in Figure 9 could also raise data management standards in the field by enabling the centralized retention of structure solution projects in maintainable storage and supporting publications with the CCP4 Cloud Archive. However, this requires substantial computational resources, with demand likely to grow as more facilities and research groups shift their MX computing operations to the Cloud.

Scalability, data policy, and security are among the key focus areas in our ongoing development of CCP4 Cloud. The service available at https://cloud.ccp4.ac.uk, maintained by the CCP4 group at RAL‐Harwell, is hosted on the STFC UK network. This infrastructure is centrally managed to industry standards, ensuring that users' data remains secure and inaccessible from external sources. STFC complies with GDPR regulations, and the UKRI privacy and data protection statement is accessible via the CCP4 Cloud homepage.

Importantly, CCP4 Cloud can be deployed independently by any scientific organization, from small research labs to large pharmaceutical companies, allowing them to meet their own privacy and data governance requirements while leveraging their local computational resources.

## 4 MATERIALS AND METHODS

CCP4 Cloud is developed using JavaScript and Python, with its source code available through GitLab (https://gitlab.com/CCP4/jsCoFE), the CCP4 repositories (https://fg.oisin.rc-harwell.ac.uk/projects/jscofe/), and the CCP4 distribution package (https://www.ccp4.ac.uk/download/). Detailed installation instructions can be found in the CCP4 Cloud documentation (https://cloud.ccp4.ac.uk/manuals/html-dev/setup.html).

The public CCP4 Cloud service, accessible at https://cloud.ccp4.ac.uk, operates on a robust infrastructure comprising 500 CPU cores, 8 GPUs (including 2 NVIDIA A100 and 6 NVIDIA RTX A6000), and 147 TB of RAID disk storage. Additionally, the DataLink service provides 60 TB of dedicated disk space, accessible to users as a “Cloud File Storage” virtual file system. CCP4 and STFC Cloud provide these resources through the IRIS framework. Resource usage—both CPU and disk—is allocated on a per‐user basis and can be adjusted upon request. There is no principal limitation on the number of users, projects or tasks in the system.

The public server is available free of charge to academic users and CCP4 license holders, offering the full range of features described in this publication.

## AUTHOR CONTRIBUTIONS

**Eugene Krissinel:** Conceptualization; software; funding acquisition; supervision; project administration; methodology; writing – review and editing; writing – original draft. **Maria Fando:** Software; writing – review and editing; validation; visualization; investigation. **Oleg Kovalevskiy:** Software; writing – review and editing; methodology; visualization; investigation; formal analysis. **Ronan Keegan:** Methodology; writing – review and editing; software. **Jools Wills:** Software; writing – review and editing; data curation; resources. **Andrey Lebedev:** Conceptualization; methodology; software; writing – review and editing. **Ville Uski:** Writing – review and editing; data curation; resources. **Charles Ballard:** Writing – review and editing; data curation; resources.

## CONFLICT OF INTEREST STATEMENT

The authors declare no conflicts of interest.

## Data Availability

The data that support the findings of this study are openly available in GitLab at https://gitlab.com/CCP4/jsCoFE.
